# Coalescing Majorana edge modes in non-Hermitian ${\mathscr{P}}{\mathscr{T}}$-symmetric Kitaev chain

**DOI:** 10.1038/s41598-020-63369-x

**Published:** 2020-04-22

**Authors:** C. Li, L. Jin, Z. Song

**Affiliations:** 1 School of Physics, Nankai University, Tianjin, 300071 China; 2 Department of Physics, University of Hong Kong, Hong Kong, China

**Keywords:** Physics, Phase transitions and critical phenomena, Topological matter

## Abstract

A single unit cell contains all the information about the bulk system, including the topological feature. The topological invariant can be extracted from a finite system, which consists of several unit cells under certain environment, such as a non-Hermitian external field. We present an exact solvable non-Hermitian finite-size Kitaev chain with ${\mathscr{P}}{\mathscr{T}}$-symmetric chemical potentials at the symmetric point. The straightforward calculation shows that there are two kinds of Majorana edge modes in this model divided by ${\mathscr{P}}{\mathscr{T}}$ symmetry-broken and unbroken. The one appeared in the ${\mathscr{P}}{\mathscr{T}}$ symmetry-unbroken region can be seen as the finite-size projection of the conventional degenerate zero modes in a Hermitian infinite system with the open boundary condition. It indicates a possible variant of the bulk-edge correspondence: The number of Majorana edge modes in a finite non-Hermitian system can be the topological invariant to identify the topological phase of the corresponding bulk Hermitian system.

## Introduction

The discovery of topological matter which exhibits topological properties in the band structure has opened a growing research field^1–5^. A particularly important concept is the bulk-edge correspondence, which indicates that, a nontrivial topological invariant in the bulk indicates localized edge modes that only appear in the presence of the open boundary in the thermodynamic limit. In general, a bulk system is constructed by stacking a great many copies of a single original unit cell in an array. In this sense, a single unit cell contains all the information about the bulk system, including the topological feature. Then the topological invariant can be, in principle, extracted from a finite system, which consists of several unit cells under certain environment, such as a non-Hermitian external field^6–8^. In fact, the original bulk-edge correspondence is an example in this context: The open boundary can be technically regarded as one of the extreme cases of adding local impurity on the bulk, that breaks the translational symmetry. On the other hand, it has been shown that a finite system with imaginary ending potentials can share the common eigenstates with an infinite system^9–11^. It implies that a finite non-Hermitian system may retain some of characteristics, such as zero-energy modes of an infinite Hermitian system. An interesting question is whether there is a generalization of the bulk-edge correspondence to non-Hermitian systems which arises from the imaginary impurity.

In this work, we investigate a non-Hermitian finite-size Kitaev ring with parity-time (${\mathscr{P}}{\mathscr{T}}$ symmetric chemical potentials. We demonstrate that the key to retrieve the Majorana zero mode from a small system is a pair of specific imaginary chemical potentials, under which the coalescing zero mode shares the identical pattern with that of the conventional zero mode in the thermodynamic limit. The coalescing zero mode in a finite-size non-Hermitian system can be directly obtained from the projection of the conventional degenerate zero modes in a Hermitian infinite system. Exact solution also shows the existence of Majorana edge modes, which emerge as a pair of ${\mathscr{P}}{\mathscr{T}}$ symmetry breaking states with imaginary eigenvalues. It indicates a variant of the bulk-edge correspondence: The number of Majorana edge modes in a finite-size non-Hermitian system can be the topological invariant to identify the topological phase of the corresponding bulk Hermitian system.

## Results

### Model hamiltonians

**Figure 1:**
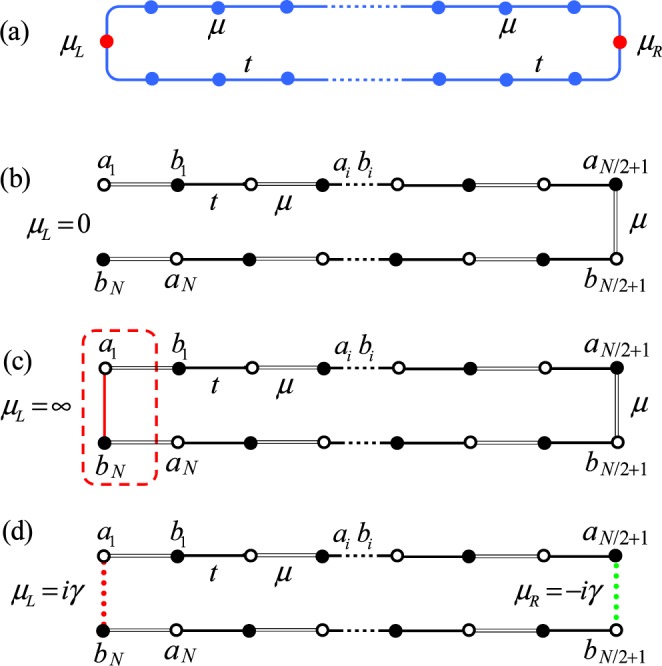
Schematic illustrations for edge modes in the Majorana Hamiltonian from Eq. (1), induced by impurities embedded in a Kitaev ring. (**a**) *N*-site Kitaev ring with uniform chemical potential *μ* and hopping amplitude (pairing amplitude) *N*. Two impurities are located at sites 1 and *N*/2 + 1, in terms of specific values of chemical potentials *μ*_*L*_ and *μ*_*R*_, respectively. The pairing term is omitted for the clarity. The corresponding Majorana lattice is a 2*N*-site dimerized ring with staggered hopping strengths *t* (single line) and *μ* (double line), which contains two specific dimers with hopping amplitudes *μ*_*L*_ and *μ*_*R*_. We focus on three cases: (**b**) *μ*_*L*_ = 0, *μ*_*R*_ = *μ*, the lattice becomes a standard 2*N*-site SSH chain, which possesses edge modes for *μ* > 1. (**c**) *μ*_*L*_ = ∞, *μ*_*R*_ = *μ*, the dimer (circled by the dashed red line) is adiabatic eliminated, and then the lattice becomes a standard 2(*N* − 1) SSH chain, which possesses edge modes for *μ* < 1. For infinite *N*, the conclusions for cases (**b**,**c**) can be regarded as the extension of original bulk-edge correspondence. (**d**) *μ*_*L*_ = −*μ*_*R*_ = *iγ*, it is ${\mathscr{P}}{\mathscr{T}}$ symmetric. For finite *N*, it has two coalescing zero modes for *μ* > 1, while two imaginary-energy modes for *μ* < 1 with two coalescing zero modes still exist. All these modes exhibit evanescent wave behavior. The number of edge modes for finite system matches the topological invariant of the bulk. It indicates that bulk-edge correspondence can be extended to the non-Hermitian regime.

We consider a one-dimensional Kitaev model with two impurities. The Hamiltonian of the tight-binding model takes the following form

1 $$\begin{array}{rcl}H & = & {H}_{0}+{H}_{{\rm{im}}},\\ {H}_{0} & = & -\mathop{\sum }\limits_{j=1}^{N}\,(t{c}_{j}^{\dagger }{c}_{j+1}+\varDelta {c}_{j}^{\dagger }{c}_{j+1}^{\dagger }+{\rm{H}}.{\rm{c}}.)-\mu \mathop{\sum }\limits_{j=1}^{N}\,(1-2{n}_{j}),\\ {H}_{{\rm{im}}} & = & (\mu -{\mu }_{L})(1-2{n}_{1})+(\mu -{\mu }_{R})(1-2{n}_{N/2+1}),\end{array}$$

where *j* is the coordinate of lattice sites and *c*_*j*_ is the fermion annihilation operator at site *j*. *H*_0_ is employed to depict *p*-wave superconductors. The hopping between (pair operator of) neighboring sites is described by the hopping amplitude *t* (the real order parameter Δ). The last term in *H*_0_ gives the chemical potential (see Fig. 1a). Imposing the periodic boundary condition *c*_1_ ≡ *c*_*N*+1_, the Hamiltonian *H*_0_ can be exactly diagonalized and the topological invariant can be obtained in various parameter regions. It provides well-known example of systems with the bulk-edge correspondence when the open boundary condition is imposed. It turns out that a sufficient long chain has Majorana modes at its two ends^12^. A number of experimental realizations of *p*-wave superconductors have found evidence for such Majorana modes^13–18^, also the realizations of Majorana modes^19–21^. Term *H*_im_ represents two impurities on the two symmetrical sites. The impurities are described in terms of specific values of chemical potentials *μ*_L_ and *μ*_R_. We note that taking *μ*_L_ = 0 or ±∞, and *μ*_R_ = *μ*, it corresponds to the open Majorana chains with different edges (see Fig. 1b,c). It is an alternative representation of bulk-boundary correspondence. In this context, the real value of *μ*_L,R_ requires an infinite system or lead to a trivial situation. In this work, we consider imaginary value of *μ*_L,R_. The imaginary potentials has been realized by the optical lattice and coupled array^22–24^ and the realization of the non-Hermitian Kitaev model in the spin model context has been detected too^25,26^. In contrast to previous studies based on Hermitian chains in the thermodynamic limit, we focus on the Kitaev model on a finite lattice system. This is motivated by the desire to get a clear physical picture of the edge mode through the investigation of a small system. We first study the present model from the description in terms of Majorana fermions. We will show that the evident indicator for phase diagram does not require infinite system.

### Majorana representation

In the following, which is showed in Fig. 1d, the impurity is taken as ${\mathscr{P}}{\mathscr{T}}$ symmetric with *μ*_L_ = *iγ* and *μ*_R_ = −*iγ*. Note that the Hamiltonian *H* is not ${\mathscr{P}}{\mathscr{T}}$ symmetric unless Δ is pure imaginary. The ${\mathscr{P}}{\mathscr{T}}$ symmetric *H* with the twisted (or open) boundary condition has been studied in^27^. Here the space-reflection operator, or the parity operator ${\mathscr{P}}$ and the time-reversal operator ${\mathscr{T}}$, are defined as

2 $${\mathscr{P}}{c}_{l}^{\dagger }{\mathscr{P}}={c}_{N+1-l}^{\dagger },{\mathscr{T}}i{\mathscr{T}}=-\,i.$$

Nevertheless, we will see that the core matrix in the Majorana fermion representation is ${\mathscr{P}}{\mathscr{T}}$ symmetric, which ensures the system is pseudo-Hermitian.

We introduce Majorana fermion operators

3 $${a}_{j}={c}_{j}^{\dagger }+{c}_{j},{b}_{j}=-i({c}_{j}^{\dagger }-{c}_{j}),$$

which satisfy the relations

4 $$\begin{array}{rcl}\{{a}_{j},{a}_{j{\prime} }\} & = & 2{\delta }_{jj{\prime} },\{{b}_{j},{b}_{j{\prime} }\}=2{\delta }_{jj{\prime} }\\ \{{a}_{j},{b}_{j{\prime} }\} & = & 0,{a}_{j}^{2}={b}_{j}^{2}=1.\end{array}$$

Then the Majorana representation of the Hamiltonian is

5 $$\begin{array}{rcl}{\mathscr{H}} & = & -\frac{i}{4}\mathop{\sum }\limits_{j=1}^{N}\,[(t+\Delta ){b}_{j}{a}_{j+1}+(t-\Delta ){b}_{j+1}{a}_{j}]\\  &  & -\,\frac{i}{2}\mathop{\sum }\limits_{j\ne 1,N/2+1}^{N}\,\mu {a}_{j}{b}_{j}+{\rm{H}}.{\rm{c}}.\\  &  & +\,\frac{\gamma }{2}({a}_{1}{b}_{1}-{a}_{N/2+1}{b}_{N/2+1}-{\rm{H}}.{\rm{c}}.).\end{array}$$

The diagonalization of *H* is directly related to a model of two coupled SSH chain, which was systematically studied in ref. ^28^.

### Edge modes

Majorana edge mode plays an important role in the characterization of the topological feature of matter, such as the bulk-edge correspondence. The Majorana particle is localized at two ends. Since the pattern of the zero mode is exponentially decaying, the bound Majorana particle must require infinite chain condition. Therefore the conventional zero mode cannot exist in a Hermitian system except for some trivial cases^29,30^. In this paper, we focus on a finite-size non-Hermitian system and we define the edge mode by the evanescent wave characterization.

We consider a Kitaev ring at the symmetric point Δ = *t*. We write down the Hamiltonian as the form

6 $${\mathscr{H}}={\psi }^{T}h\psi ,$$

in the basis *ψ*^*T*^ = (*a*_1_, *b*_1_, *a*_2_, *b*_2_, *a*_3_, *b*_3_, …, *a*_*N*_, *b*_*N*_), where *h* represents a 2*N* × 2*N* matrix. Here matrix *h* is explicitly written as

7 $$\begin{array}{rcl}h & = & -\frac{i}{2}(\mathop{\sum }\limits_{l=1}^{N}\,t|2l\rangle \langle 2l+1|+\mathop{\sum }\limits_{l\ne 1,N/2+1}^{N}\,\mu |2l-1\rangle \langle 2l|+{\rm{H}}.{\rm{c}}.)\\  &  & +\,\frac{\gamma }{2}(|1\rangle \langle 2|-|N+1\rangle \langle N+2|-{\rm{H}}.{\rm{c}}.)\end{array}$$

where basis $\{|j\rangle ,j\in [1,2N]\}$ is an orthonormal complete set, $\langle \,j|j{\prime} \rangle ={\delta }_{jj{\prime} }$. By taking the linear transformation

8 $$\{\begin{array}{r}|\sigma ,2l-1\rangle =\frac{{e}^{-i\pi /4}}{2}(|2l\rangle +i\sigma |2N+3-2l\rangle )\\ |\sigma ,2l\rangle =\frac{{e}^{i\pi /4}}{2}(|2l+1\rangle -i\sigma |2N+2-2l\rangle )\end{array}$$

with *l* ∈ [1, *N*/2] and *σ* = ±, we can express matrix *h* as *h* = *h*_+_ + *h*_*−*_ with

9 $$[{h}_{+},{h}_{-}]=0,$$

where

10 $$\begin{array}{rcl}{h}_{\sigma } & = & t\mathop{\sum }\limits_{l=1}^{N/2}\,|\sigma ,2l-1\rangle \langle \sigma ,2l|-\mu \mathop{\sum }\limits_{j=1}^{N/2-1}\,|\sigma ,2l\rangle \langle \sigma ,2l+1|\\  &  & +\,{\rm{H}}.{\rm{c}}.+\sigma i\gamma (|\sigma ,1\rangle \langle \sigma ,1|-|\sigma ,N\rangle \langle \sigma ,N|).\end{array}$$

Obviously, *h*_±_ describes two identical SSH chains with opposite imaginary ending potentials ±*iγ*. It is a ${\mathscr{P}}{\mathscr{T}}$ symmetric system and has been studied in refs. ^31^ for *μ* > *t* and the topological phase in the similar systems has been studied in refs. ^32^. It is shown that there is a single zero mode in such a finite degree of non-Hermiticity at the exceptional point (EP).

Now we concentrate on a single non-Hermitian SSH chain with the Hamiltonian

11 $$\begin{array}{rcl}{h}_{{\rm{SSH}}} & = & \mathop{\sum }\limits_{l=1}^{N/2}\,|2l-1\rangle \langle 2l|-\mathop{\sum }\limits_{l=1}^{N/2-1}\,\mu |2l\rangle \langle 2l+1|\\  &  & +\,{\rm{H}}.{\rm{c}}.+i\gamma (|1\rangle \langle 1|-|N\rangle \langle N|),\end{array}$$

where we take *t* = 1 and *μ* = 0 for the sake of simplicity. Based on the commutation relation between *h*_+_ and *h*_−_, we can use *h*_SSH_ $({h}_{{\rm{SSH}}}^{\dagger })$ to study the properties of *h*_+_ (*h*_−_). But one should attention that it does not means *h*_SSH_ $({h}_{{\rm{SSH}}}^{\dagger })={h}_{+}$ (*h*_**−**_). Here we redefine the space-reflection operator, or the parity operator ${\mathscr{P}}$ in the space spanned by the complete set {|*l*〉}. The action ${\mathscr{P}}$ on |*l*〉 is given by the equality

12 $${\mathscr{P}}|l\rangle =|N+1-l\rangle .$$

Then we have $[{h}_{{\rm{SSH}}},{\mathscr{P}}{\mathscr{T}}]=0$, which implies that *h*_SSH_ is pseudo-Hermitian^33^, i.e., *h*_SSH_ has either real spectrum or its complex eigenvalues occur in complex conjugate pairs. On the transition from a pair of real levels to a complex conjugate pair, or EP, two levels coalesce to a single level. The key point of our approach is to link the zero-eigenvalue coalescing eigenvector to the conventional Majorana zero mode in the thermodynamic limit.

The Bethe ansatz wave function $|{\psi }_{k}\rangle ={\sum }_{l=1}^{N}\,{f}_{l}^{k}|l\rangle$ has the form

13 $${f}_{l}^{k}=\{\begin{array}{ll}{A}_{k}{e}^{ikl}+{B}_{k}{e}^{-ikl}, & l=2j-1\\ {C}_{k}{e}^{ikl}+{D}_{k}{e}^{-ikl}, & l=2j\end{array},$$

where *j* = 1, 2, …, *N*/2. Following the derivation in the Method C, if we take the ***μ***_***L***_ = ***iγ*** and ***μ***_**R**_** = −*iγ***, the appearance of the zero mode requires

14 $$\gamma ={\mu }^{1-N/2},$$

and based on the specific form above, there are three types of eigenvector |***ψ***_***k***_**〉, with the eigenvalue (see details in the Method A)**

15 $${\varepsilon }_{k}=\pm \sqrt{1+{\mu }^{2}-\mu ({e}^{2ik}+{e}^{-2ik})}.$$

In general, the EP varies as the system size changes. In the present model, *γ* = *μ*^1−*N*/2^ is *N* dependent except when *μ* = 1. It is reasonable that the system is always at the EP for any *N*. We note that in the case of *μ* = 1, it reduces to a uniform chain with *γ* = 1, which was studied in ref. ^34^. The solutions for *μ* ≠ 1 is concluded as follows.

(i) Scattering vector with real eigenvalues: In this case, *k* is a real number, the eigenvalue is real. The energy gap has a lower bound

16 $${\Delta }_{{\rm{Gap}}}\ge 2|1-\mu |,$$

which is crucial to protect the degenerate ground states of the original Kitaev model from decoherence.

(ii) Coalescing vector with zero eigenvalue: Here the wave vector is imaginary, $k=\pm \frac{i}{2}\,\mathrm{ln}\,\mu$. The eigenvector has the form

17 $$|{\psi }_{{\rm{zm}}}\rangle =\Omega \mathop{\sum }\limits_{j=1}^{N/2}\,({\mu }^{1-j}|2j-1\rangle -i{\mu }^{j-N/2}|2j\rangle ),$$

satisfying

18 $${h}_{{\rm{SSH}}}|{\psi }_{{\rm{zm}}}\rangle =0.$$

where $\Omega ={\mu }^{N\mathrm{/2}-1}\sqrt{(1-{\mu }^{2})/(2-2{\mu }^{N})}$ is the Dirac normalizing constant. It is ${\mathscr{P}}{\mathscr{T}}$ symmetric and has a zero biorthogonal norm, indicating the coalescence of two levels. Actually, the zero-mode vector for ${h}_{{\rm{SSH}}}^{\dagger }$ can be constructed as

19 $$|{\eta }_{{\rm{zm}}}\rangle =\Omega \mathop{\sum }\limits_{j=1}^{N/2}\,({\mu }^{1-j}|2j-1\rangle +i{\mu }^{j-N/2}|2j\rangle ),$$

satisfying

20 $${h}_{{\rm{SSH}}}^{\dagger }|{\eta }_{{\rm{zm}}}\rangle =0.$$

On the other hand, it is easy to check

21 $$\langle {\eta }_{{\rm{zm}}}|{\psi }_{{\rm{zm}}}\rangle =0,$$

and

22 $$|{\eta }_{{\rm{zm}}}\rangle =i{\mathscr{P}}|{\psi }_{{\rm{zm}}}\rangle \,{\rm{or}}\,|{\eta }_{{\rm{zm}}}\rangle ={(|{\psi }_{{\rm{zm}}}\rangle )}^{\ast },$$

which indicate that the vector |*ψ*_zm_〉 has a zero biorthogonal norm and the relations between two conjugate vectors. Based on these facts we conclude that the zero-mode vector |*ψ*_zm_〉 is a coalescing vector for *μ* ≠ 1. The exact wave function of |*ψ*_zm_〉 clearly indicates that it is a superposition of two parts with nonzero amplitudes only located at even or odd sites, respectively. We will show that vectors |*η*_zm_〉 and |*ψ*_zm_〉 have a close relation to the standard zero modes of a Hermitian chain in the thermodynamic limit. Here, the real value of *μ*_L,R_ can still lead to two zero modes, but they are two degenerate states and with only one phase exists, no matter

(iii) Evanescent wave vector with imaginary eigenvalue: The derivation in the Method A shows that this kind of state only appears at *μ* < 1. In this case, *k* is still imaginary. Since matrix *h*_SSH_ is pseudo-Hermitian, this type of eigenvector always appears in pair. The wave vector approximately takes

23 $$k=\pm i\frac{N-1}{2}\,\mathrm{ln}\,\mu $$

in large *N* or small *μ* and the wave function reads

24 $$|{\psi }_{{\rm{IM}}}^{\sigma }\rangle \approx \frac{1}{2}[(1+\sigma )|1\rangle +(1-\sigma )|N\rangle ],$$

satisfying

25 $${h}_{{\rm{SSH}}}|{\psi }_{{\rm{IM}}}^{\sigma }\rangle =i\sigma |{\varepsilon }_{{\rm{IM}}}||{\psi }_{{\rm{IM}}}^{\sigma }\rangle .$$

with approximate eigenvalue

26 $${\varepsilon }_{{\rm{IM}}}\approx \pm i{\mu }^{1-N/2},$$

**Figure 2:**
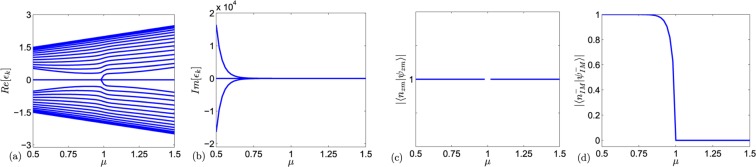
Numerical calculation of the Hamiltonian (11) when *N* = 30. Plots (**a**,**b**) show the real part and imaginary part of eigenvalues, the zero mode exists for all *μ* value and the imaginary edge mode only exists when *μ* < 1. Plots (**c**,**d**) show the fidelity $|\langle {n}_{zm}|{\psi }_{zm}\rangle |$ and $|\langle {n}_{IM}^{-}|{\psi }_{IM}^{-}\rangle |$, where $\langle {n}_{zm}|$ and $\langle {n}_{IM}^{-}|$ means the numerically calculated zero mode and one of the imaginary edge mode, respectively.

We can see that the ${\mathscr{P}}{\mathscr{T}}$ symmetry is broken. Both the two vectors present evanescent wave and

27 $$|{\psi }_{{\rm{IM}}}^{\sigma }\rangle ={\mathscr{P}}{\mathscr{T}}|{\psi }_{{\rm{IM}}}^{-\sigma }\rangle ,$$

which indicates that $|{\psi }_{{\rm{IM}}}^{\pm }\rangle$ are symmetry breaking. We summarize the solutions in Table 1 and present the numerical calculation of energy-levels of Hamiltonian (11) and the fidelity $\langle {n}_{{\rm{IM}}}^{-}|{\psi }_{{\rm{IM}}}^{-}\rangle$ and $\langle {n}_{{\rm{zm}}}|{\psi }_{{\rm{zm}}}\rangle$ respectively in Fig. 2, which shows the effectiveness of our analytical solution.

**Table 1:** For *N*-site system, *γ* = *μ*^1−*N*/2^, *n*_I_ is the number of imaginary levels, *n*_EP_ is the number of coalescing state, *n*_S_ is the number of real levels. We have *n*_I_ + 2*n*_EP_ + *n*_S_ = *N*.

*μ*	*n*_I_	*n*_EP_	*n*_S_	Symmetry
*μ* > 1	0	1	*N* − 2	${\mathscr{P}}{\mathscr{T}}-{\rm{unbroken}}$
*μ* < 1	2	1	*N* − 4	${\mathscr{P}}{\mathscr{T}}-{\rm{broken}}$

We would like to point out that the real value of *μ*_L,R_ can still produce two zero modes (or just two eigenstates with zero energy) once they satisfy one condition (showed in the Method), but they are two degenerate states and with only one phase exists, no matter *μ* > 1 or <1. It leads to a trivial model without symmetry breaking and coalescing even if there are some connections between the finite zero modes and conventional zero modes of kitaev model. As we mentioned before, there are also researches focus on the ${\mathscr{P}}{\mathscr{T}}$ symmetric *H* with the twisted (or open) boundary condition where the similar edge modes can be found^27^, but with totally different topological meaning according to their system conditions. For a more general quasi-1D kitaev chain with *p*_*x*_ + *ip*_*y*_ superconducting pairing^18^, Majorana zero modes emerge in such an open system without fine-tuning as a result of charge-conjugation symmetry, and Majorana dark state (MDS) can appear too.

It is well known that the number of Majorana zero mode for an infinite Hermitian Kitaev chain with open boundary conditions can be a topological invariant, referred as the bulk-edge correspondence. Similarly, the result in Table 1 indicates the number of Majorana edge modes in a finite non-Hermitian system can also be the topological invariant to identify the topological phase of the corresponding bulk Hermitian system. In this situation, two Majorana edge modes appear as a pair of ${\mathscr{P}}{\mathscr{T}}$ symmetry breaking states with imaginary eigenvalues, this can be regarded as a variant of the bulk-edge correspondence in the complex regime. Recently, non-Hermitian SSH chains were experimentally realized by coupled dielectric microwave resonators^35,36^ and photonic lattices^37,38^. In Method B, we provide exact solutions for a 6-site system to demonstrate our main idea, which can be a protocol for the experimental investigation. Before, many works showed the connection between the Majorana zero modes (edge modes) and the topological properties of the Kitaev model in general condition (Δ ≠ *t*) with the numerical simulation^39–41^, which give the phase diagram and indicate the difficulty of exact solution. Here, we only consider the kitaev ring on the symmetric point Δ = *t*, this simple situation is good enough to give a clear physical picture and solution to show that how the appearance of non-zero *μ*_*R*_ and *μ*_*L*_ change the topology of the system and how the edge modes (zero modes) connects with conventional ones in a large *N* limit.

### Connection to conventional zero mode

In this section, we investigate the connection for the zero-mode states between the present non-Hermitian model and infinite Hermitian SSH chain. We consider *μ*_SSH_ in the large *N* limit and analyze the solutions in the following two regions.

(i) In the case of *μ* > 1, we have *γ* = *μ*^1−*N*/2^ → 0. Matrices *h*_SSH_ and ${h}_{{\rm{SSH}}}^{\dagger }$ become the same matrix of *N* -site single-particle Hermitian SSH chain with open boundary conditions. Two zero-mode wave functions |*η*_zm_〉 and |*ψ*_zm_〉 become two degenerate zero modes of the same Hermitian SSH chain. Remarkably, the Eq. (21) for characterizing the coalescing levels is nothing but the Dirac orthogonality of two degenerate zero modes. The coalescing zero mode is the finite-size projection of the conventional degenerate zero modes in a Hermitian infinite system with the open boundary condition. In this sense, the coalescing zero modes |*η*_zm_〉 and |*ψ*_zm_〉 for any small *N* carry the complete information of conventional zero modes for the SSH chain.

(ii) Now we turn to the case of *μ* < 1. In contrast to the case (i), we have *γ* = *μ*^1−*N*/2^ ≫ 1. Therefore, two ending sites are adiabatically eliminated from the *N*-site chain. The original system is separated into three independent parts. Two ending-site parts possess two eigenvectors with imaginary eigenvalues ±*iγ*. The third part corresponds to an (*N* − 2)-site single-particle Hermitian SSH chain with open boundary conditions. Although an SSH chain is at total different topological phases for *μ* > 1 and *μ* < 1 respectively. However, such an (*N* − 2)-site Hermitian SSH chain is the same as that of the *N*-site one in the large *N* limit. In this sense, the coalescing zero mode for *μ* < 1 represents the same feature as that of *μ* > 1.

**Figure 3:**
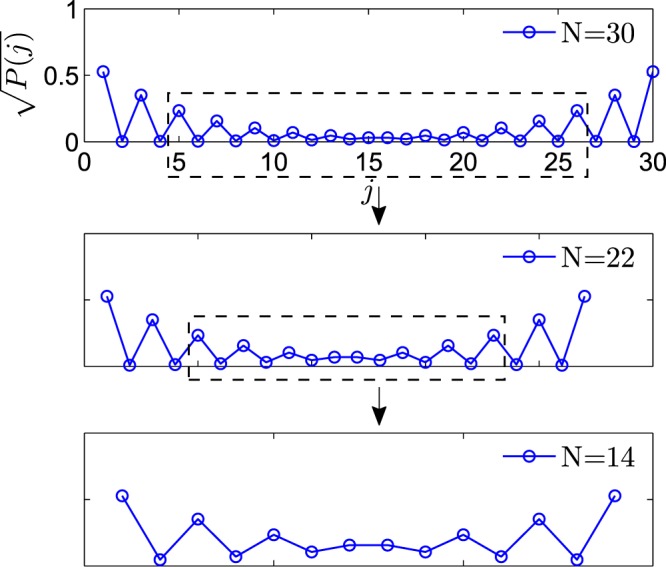
Plots of Dirac norm distribution of the coalescing zero mode from Eq. (17) for the Hamiltonian (11) with *μ* = 1.5 *γ* = *μ*^1−*N*/2^ for different *N*. The dashed boxes indicate that the plot for *N* = 22 is a part of *N* = 30, while the plot of *N* = 14 is a part of *N* = 22. Three systems share a common part of wave vector.

We plot Dirac norm distribution of the coalescing zero mode from Eq. (17) for the Hamiltonian (11) with *N* = 30, 22, and 14, where

28 $$P(j)=|\langle j|{\psi }_{{\rm{zm}}}\rangle |=\{\begin{array}{l}\sqrt{\frac{2(1-{\mu }^{N})}{1-{\mu }^{2}}}{\mu }^{N/2-l},j=2l-1,\\ \sqrt{\frac{2(1-{\mu }^{N})}{1-{\mu }^{2}}}{\mu }^{l-1},j=2l,\end{array},l=1,2,\ldots ,N/2,$$

as the demonstration of our main result (see Fig. 3). We see that systems with different size share a common part of wave vector. It indicates that one can retrieve the information of the Majorana zero mode in the thermodynamic limit from a small non-Hermitian system. Two degenerate conventional zero modes corresponds to the left and right vectors of the coalescing zero mode.

## Discussion

In conclusion, we have demonstrated a connection between the topological characterization of an infinite system and the property of a specific small system through a concrete Kitaev model. We have shown that a fine-tuned non-Hermitian ${\mathscr{P}}{\mathscr{T}}$-symmetric chemical potential in the finite-size Kitaev ring results in coalescing Majorana zero mode. In particular, such a zero mode in small lattice contains all the information of the conventional degenerate Majorana zero modes in the thermodynamic limit. In addition, the number of edge modes, which includes a pair modes with imaginary eigenvalues, can be a topological invariant to characterize the quantum phase diagram of the corresponding bulk Hermitian system. Although the obtained conclusion is only based on a specific model, it may be a universal feature of the topological system. The recent studies of non-Hermitian Dirac superconductor have shown this point^42,43^. The underlying mechanism is two fold. Firstly, a single unit cell contains all the information about the bulk system, including the topological feature. Secondly, a finite-size non-Hermitian system, in some way, can be regarded as a sub-system embedded in an infinite system.

## Methods

### General evanescent solution for non-hermitian ssh chain

In this section, we provide the evanescent solutions of the Hamiltonian (11) with *γ* < *μ*^1−*N*/2^ by the Bethe ansatz method. The Bethe ansatz wave function has the form

29 $${f}_{l}^{k}=\{\begin{array}{ll}{A}_{k}{e}^{ikl}+{B}_{k}{e}^{-ikl}, & l=2j-1\\ {C}_{k}{e}^{ikl}+{D}_{k}{e}^{-ikl}, & l=2j\end{array},$$

where *j* = 1, 2, …, *N*/2. The explicit form of Schrodinger equation

30 $$H|{\psi }_{k}\rangle ={\varepsilon }_{k}|{\psi }_{k}\rangle $$

is expressed as

31 $$\{\begin{array}{rcl}{f}_{m-1}^{k}-\mu {f}_{m+1}^{k} & = & {\varepsilon }_{k}{f}_{m}^{k}\\ {f}_{m}^{k}-\mu {f}_{m-2}^{k} & = & {\varepsilon }_{k}{f}_{m-1}^{k}\\ {f}_{m+2}^{k}-\mu {f}_{m}^{k} & = & {\varepsilon }_{k}{f}_{m+1}^{k}\\ {f}_{m+1}^{k}-\mu {f}_{m+3}^{k} & = & {\varepsilon }_{k}{f}_{m+2}^{k}\end{array}$$

in the bulk and

32 $$\{\begin{array}{rcl}i\gamma {f}_{1}^{k}+{f}_{2}^{k} & = & {\varepsilon }_{k}{f}_{1}^{k}\\ {f}_{N-1}^{k}-i\gamma {f}_{N}^{k} & = & {\varepsilon }_{k}{f}_{N}^{k}\end{array}$$

at two ends, where *m* = 2*j*, *j* = 1, 2, 3, …, *N*/2. From Eq. (31) we have the spectrum

33 $${\varepsilon }_{k}=\pm \sqrt{1+{\mu }^{2}-\mu ({e}^{2ik}+{e}^{-2ik})},$$

and coefficients

34 $$\frac{{B}_{k}}{{D}_{k}}=\frac{{C}_{k}}{{A}_{k}}={e}^{-ik}\sqrt{\frac{1-\mu {e}^{2ik}}{1-\mu {e}^{-2ik}}}.$$

Together with Eq. (32), we get the equation about *k*

35 $$\begin{array}{c}({\varepsilon }_{k}^{2}-{\gamma }^{2}-1)[{e}^{i(N-2)k}-{e}^{-i(N-2)k}]\\ +\,\mu {e}^{i(N-4)k}-{e}^{-i(N-4)k}]+\mu ({\gamma }^{2}+{\varepsilon }_{k}^{2})({e}^{ikN}-{e}^{-ikN})\\ =\,0.\end{array}$$

In general, the wave function |*ψ*_*k*_〉 with real *k* always represents the scattering vector. Since the real *k* is bounded by ±*π*, the gap between two branches of eigenvalues *ε*_*k*_ is also bounded by 2|1 − *μ*|.

We are interested in the evanescent wave solution which corresponds to *k* = *iκ* or *π* + *iκ* (*κ* is a real number). In this case, Eq. (35) becomes

36 $$\begin{array}{c}({\varepsilon }_{k}^{2}-{\gamma }^{2}-1)\sinh \,[(N-2)\kappa ]+\mu \,\sinh \,[(N-4)\kappa ]\\ \,+\,\mu ({\gamma }^{2}+{\varepsilon }_{k}^{2})\sinh (N\kappa )=0,\end{array}$$

with eigenvalue

37 $${\varepsilon }_{k}=\pm \sqrt{1+{\mu }^{2}-2\mu \,\cosh (2\kappa )}.$$

At first, it is not hard to find that there are always two solutions

38 $$\kappa =\pm \frac{1}{2}\,\mathrm{ln}\,\mu $$

for Eq. (36). It leads to *A*_*k*_ = *D*_*k*_ = 0 with $\kappa =-\,\frac{1}{2}\,\mathrm{ln}\,\mu$ (or *B*_*k*_ = *C*_*k*_ = 0 with $\kappa =\frac{1}{2}\,\mathrm{ln}\,\mu$) according to Eq. (34). This solution holds for all value of *μ* ≠ 1. Secondly, in the case of

39 $${e}^{-\kappa N},{e}^{-\kappa (N-2)},{e}^{-\kappa (N-4)}\ll 1,$$

Equation (36) can be reduced to

40 $$\begin{array}{c}({\varepsilon }_{k}^{2}-{\gamma }^{2}-1){e}^{\kappa (N-\mathrm{2)}}+\mu {e}^{\kappa (N-\mathrm{4)}}\\ +\,\mu ({\gamma }^{2}+{\varepsilon }_{k}^{2}){e}^{\kappa N}=\mathrm{0,}\end{array}$$

or a more popular form

41 $${e}^{-4\kappa }+\frac{{\varepsilon }_{k}^{2}-{\gamma }^{2}-1}{\mu }{e}^{-2\kappa }+({\gamma }^{2}+{\varepsilon }_{k}^{2})=0.$$

Submitting the expression of *ε*_*k*_ into the equation above, it can be approximately reduced to a linear equation for *e*^−2*κ*^, which has the solution

42 $$\kappa =\frac{1-N}{2}\,\mathrm{ln}\,\mu .$$

We note that the relations

43 $$\{\begin{array}{rcl}{\kappa }_{\mu =1} & = & 0\\ \frac{\partial \kappa }{\partial \mu } &  <  & 0\end{array},$$

ensure the condition in Eq. (39) can be satisfied for the region *μ* < 1. Then the obtained solution is justified. A similar procedure can be performed in the case of ${e}^{\kappa N},{e}^{\kappa (N-2)},{e}^{\kappa (N-4)}\ll 1$. In summary, a pair of solutions with imaginary wave vectors are

44 $$k=\pm i\frac{1-N}{2}\,\mathrm{ln}\,\mu .$$

And the corresponding eigenvalues are

45 $${\varepsilon }_{{\rm{IM}}}\approx \pm i{\mu }^{1-N/2}.$$

### Example solution for *N* = 6

We demonstrate the above analysis via exact solutions for *N* = 6 system. Taking *μ* = 2, we have *γ* = 1/4, matrix *h*_SSH_ is expressed explicitly as

46 $${M}_{1}=(\begin{array}{cccccc}i/4 & 1 & 0 & 0 & 0 & 0\\ 1 & 0 & 2 & 0 & 0 & 0\\ 0 & 2 & 0 & 1 & 0 & 0\\ 0 & 0 & 1 & 0 & 2 & 0\\ 0 & 0 & 0 & 2 & 0 & 1\\ 0 & 0 & 0 & 0 & 1 & -i/4\end{array}).$$

The eigenvalues are

47 $$\begin{array}{lllll}{\varepsilon }_{1} & = & {\varepsilon }_{2} & = & 0,\\ {\varepsilon }_{3} & = & -{\varepsilon }_{4} & = & \frac{1}{8}\sqrt{350+2\sqrt{3553}},\\ {\varepsilon }_{5} & = & -{\varepsilon }_{6} & = & \frac{1}{8}\sqrt{350-2\sqrt{3553}},\end{array}$$

which are all real. Two zero-mode eigen vectors are identical, i.e.,

48 $${\phi }_{1}={\phi }_{2}=(4i,1,-2i,-2,i,4),$$

which has zero biorthogonal norm.

Taking *μ* = 1/2, we have *γ* = 4, matrix *h*_SSH_ is expressed explicitly as

49 $${M}_{2}=(\begin{array}{cccccc}i4 & 1 & 0 & 0 & 0 & 0\\ 1 & 0 & 1/2 & 0 & 0 & 0\\ 0 & 1/2 & 0 & 1 & 0 & 0\\ 0 & 0 & 1 & 0 & 1/2 & 0\\ 0 & 0 & 0 & 1/2 & 0 & 1\\ 0 & 0 & 0 & 0 & 1 & -i4\end{array}),$$

The eigenvalues are

50 $$\begin{array}{lllll}{\varepsilon }_{1} & = & {\varepsilon }_{2} & = & 0,\\ {\varepsilon }_{3} & = & -{\varepsilon }_{4} & = & \frac{i}{2}\sqrt{2\sqrt{238}+25},\\ {\varepsilon }_{5} & = & -{\varepsilon }_{6} & = & \frac{1}{2}\sqrt{2\sqrt{238}-25},\end{array}$$

which contains a pair of imaginary numbers. Two zero-mode eigen vectors are identical, i.e.

51 $${\phi }_{1}={\phi }_{2}=(i,4,-2i,-2,4i,1),$$

which has zero biorthogonal norm. In both two cases, vectors *ϕ*_1_, *ϕ*_2_ are not normalized.

### The condition of zero modes appear

For a general situation, the Hamiltonian (11) can be rewritten as

52 $$\begin{array}{rcl}{h}_{{\rm{SSH}}} & = & \mathop{\sum }\limits_{l=1}^{N/2}\,|2l-1\rangle \langle 2l|-\mathop{\sum }\limits_{l=1}^{N/2-1}\,\mu |2l\rangle \langle 2l+1|\\  &  & +\,{\rm{H}}.{\rm{c}}.+{\mu }_{{\rm{L}}}|1\rangle \langle 1|+{\mu }_{{\rm{R}}}|N\rangle \langle N|.\end{array}$$

The eigenvalue equation corresponding to this Hamiltonian can be seen as the coupled pair of equations

53 $$\begin{array}{rcl}-\mu {\phi }_{2n}+{\phi }_{2n+2} & = & E{\phi }_{2n+1}\\ {\phi }_{2n-1}-\mu {\phi }_{2n+1} & = & E{\phi }_{2n},\end{array}$$

with *n* = 1, …, (*N*/2) − 1 together with the boundary conditions

54 $$\begin{array}{rcl}{\mu }_{{\rm{L}}}{\phi }_{1}+{\phi }_{2} & = & E{\phi }_{1},\\ {\mu }_{{\rm{R}}}{\phi }_{N}+{\phi }_{N-1} & = & E{\phi }_{N}.\end{array}$$

When *E* = 0, it is not hard to find that

55 $$\begin{array}{ll}{\phi }_{2n+2} & =\,\mu {\phi }_{2n},{\phi }_{2n+1}=\,\frac{1}{\mu }{\phi }_{2n-1},\\ {\phi }_{2} & =\,-{\mu }_{{\rm{L}}}{\phi }_{1},{\phi }_{N}=-\frac{1}{{\mu }_{{\rm{R}}}}{\phi }_{N-1},\end{array}$$

suppose *ϕ*_1_ = *B*, *ϕ*_2_ = *A*, the equations above give

56 $$\begin{array}{ll}{\phi }_{2n} & =\,A{\mu }^{n-1},{\phi }_{2n-1}=B\frac{1}{{\mu }^{n-1}},\\ A & =\,-{\mu }_{{\rm{L}}}B,A{\mu }^{N/2-1}=-\frac{1}{{\mu }_{{\rm{R}}}}B\frac{1}{{\mu }^{N/2-1}},\end{array}$$

that means once *E* = 0, the two on-site potentials have to satisfy

57 $${\mu }_{{\rm{L}}}{\mu }_{{\rm{R}}}=\frac{1}{{\mu }^{N-2}}$$
